# Association of environmental exposure to chromium with differential DNA methylation: An epigenome-wide study

**DOI:** 10.3389/fgene.2022.1043486

**Published:** 2023-01-04

**Authors:** Meiduo Zhao, Jingtao Wu, Jing Xu, Ang Li, Yayuan Mei, Xiaoyu Ge, Guohuan Yin, Xiaolin Liu, Lanping Wei, Qun Xu

**Affiliations:** ^1^ Department of Epidemiology and Biostatistics, Institute of Basic Medical Sciences Chinese Academy of Medical Sciences, School of Basic Medicine Peking Union Medical College, Beijing, China; ^2^ Center of Environmental and Health Sciences, Chinese Academy of Medical Sciences, Peking Union Medical College, Beijing, China; ^3^ Department of Epidemiology and Biostatistics, Jinzhou Medical University, Jinzhou, Liaoning, China; ^4^ Jinzhou Central Hospital, Jinzhou, Liaoning, China

**Keywords:** chromate, DNA methylation, epigenome-wide association study, Illumina 850K, epigenetics

## Abstract

**Introduction:** Previous studies have reported that chromium (Cr)-induced epigenetic alterations and DNA methylation play a vital role in the pathogenesis of diseases induced by chromium exposure. Epigenomic analyses have been limited and mainly focused on occupational chromium exposure; their findings are not generalizable to populations with environmental Cr exposure.

**Methods:** We identified the differential methylation of genes and regions to elucidate the mechanisms of toxicity related to environmental chromium exposure. DNA methylation was measured in blood samples collected from individuals in Cr-contaminated (*n* = 10) and unexposed areas (*n* = 10) by using the Illumina Infinium HumanMethylation850K array. To evaluate the relationship between chromium levels in urine and CpG methylation at 850 thousand sites, we investigated differentially methylated positions (DMPs) and differentially methylated regions (DMRs) by using linear models and DMRcate method, respectively. The model was adjusted for biologically relevant variables and estimated cell-type compositions.

**Results:** At the epigenome-wide level, we identified five CpGs [cg20690919 (*p*_FDR =_0.006), cg00704664 (*p*_FDR =_0.024), cg10809143 (*p*_FDR =_0.043), cg27057652 (*p*_FDR =_0.047), cg05390480 (*p*_FDR =_0.024)] and one DMR (chr17: 19,648,718-19,648,972), annotated to *ALDH3A1* genes (*p* < 0.05) as being significantly associated with log_2_ transformed urinary chromium levels.

**Discussion:** Environmental chromium exposure is associated with DNA methylation, and the significant DMPs and DMR being annotated to cause DNA damage and genomic instability were found in this work. Research involving larger samples is required to further explore the epigenetic effect of environmental chromium exposure on health outcomes through DNA methylation.

## 1 Introduction

Chromium (Cr), a major source of heavy metal pollution, is frequently used in industrial products and processes, resulting in high levels of Cr exposure. Its redox form, hexavalent chromium Cr (VI), is classified as a type I carcinogen by the International Agency for Research on Cancer (Wilbur et al., 2012). Cr (VI) is rarely found in nature and is mainly produced through electroplating, stainless steel welding, leather production, and the production of Cr-containing pigments and paints (Martinez-Zamudio and Ha, 2011; Santonen et al., 2019). Cr (VI) is a recognized human carcinogen, but the mechanisms underlying Cr (VI)-induced carcinogenesis are not fully understood (Abreu et al., 2018; Feng et al., 2020).

Studies investigating cancer biomarkers have discovered that certain gene expression profiles, epigenetic signatures, heterozygosity losses, and allelic imbalances result from cancer development (Kothari et al., 2018; Marino et al., 2022). Of the molecular processes associated with cancer, DNA methylation plays an important role in early cancer development through epigenetic reprogramming (Cantone and Fisher, 2013) and is a mechanism underlying the effects of environmental pollutants on human health (Chervona et al., 2012; Brocato and Costa, 2013; Tiffon, 2018). Researchers found that metal carcinogens do not cause many mutations or have strong genotoxic effects; instead, they induce various epigenetic changes (Wang and Yang, 2019). Cr-induced epigenetic alterations have received increased attention and been recognized as having promising potential as novel biomarkers for cancer prognostic prediction and early cancer diagnosis (Laird, 2003).

Increasingly, *in vitro*, and epidemiological studies have highlighted that Cr exposure can affect human health by regulating DNA methylation. Studies have provided evidence that Cr changes global DNA methylation (Lou et al., 2013; Dworzański et al., 2020; Ha et al., 2021) and locus-specific DNA methylation (Hu et al., 2018; Feng et al., 2020; Xu et al., 2021). Ha et al. (2021) demonstrated that global DNA methylation was reduced in workers exposed to chromate and that programmed cell death 5 (*PDCD5*) percentage was significantly associated with global DNA hypomethylation. A study (Lou et al., 2013) revealed that high levels of Cr (VI) compound exposure could result in global DNA hypomethylation in the human B lymphoblastoid cell line (HMy2.CIR) or human lung cancer cell line (A549). Additionally, the hypermethylation of *MGMT*, *HOGG1*, and *RAD51* caused by Cr exposure was reported in a population-based study (Hu et al., 2018). Xu et al. (2021) demonstrated that Cr (VI) exposure can regulate *neurogenin1* and *neurod1* gene expression through DNA hypomethylation, which leads to neurotoxicity. However, few epidemiological studies have investigated differential DNA methylation in response to Cr exposure at the epigenome-wide level.

Although Cr-induced DNA methylation changes have been reported, little evidence of the effects of high levels of Cr in the environment (caused by industrial pollution) on DNA methylation is available. Research has not confirmed whether Cr can result in novel differential DNA methylation locus under long-term environmental exposure; most research has focused on occupational exposure (Hu et al., 2018; Feng et al., 2020; Ha et al., 2021) or chromate-related lung cancer (Kondo et al., 2006; Tsuboi et al., 2020), and the results are not generalizable. Researchers should fully explore the effects of Cr exposure on human health. We applied the Illumina Infinium HumanMethylation850K BeadChip (Illumina Inc, United States) to measure DNA methylation levels at more than 850,000 CpG sites and investigated epigenetic changes between groups with and without environmental Cr exposure. We conducted a cross-sectional study by using data from Jinzhou City in the People’s Republic of China, an area with high levels of Cr, to explore the associations between Cr exposure and differentially methylated positions (DMPs) and differentially methylated regions (DMRs) and to elucidate the long-term health effects of high levels of environmental Cr exposure.

## 2 Materials and methods

### 2.1 Study design

To reveal epigenome-wide DNA methylation patterns in response to Cr exposure, a cross-sectional study was conducted in Taihe district, Jinzhou City, Liaoning Province of northeast China, which experienced severe Cr pollution in the 1960s (Zhao et al., 2022). Because of the large-scale production of ferroalloys, large amounts of wastewater, ore residue, and gas containing Cr (VI) were discharged into the environment surrounding Taihe district. Although the local government has taken restorative measures, the local environment experienced severe Cr (VI) pollution, and relatively high levels of Cr remain (Yong-Gang et al., 2019). Participants are included in the present study, who have lived in local areas for more than 40 years without occupational Cr exposure or the history of cancers. Finally, the Cr exposure group comprised 10 individuals from Cr-exposed regions along a contaminated river less than 10 km from the ferroalloy factory, and the unexposed group comprised 10 individuals from unexposed regions at least 50 km from that factory (Xu et al., 2018). Both groups had similar lifestyles, cultural backgrounds, and socioeconomic status. All participants signed informed consent forms.

### 2.2 Sample collection, processing, and analysis

Urine specimens were collected in heavy metal–free plastic tubes (Falcon, Thermo Fisher Scientific, Waltham, Massachusetts, United States). The tubes containing these samples were transferred to the laboratory in foam boxes with dry ice and stored at −80°C for later analysis. The concentrations of Cr in urine were determined through inductively coupled plasma mass spectrometry (ICP-MS; Thermo Fisher X2; Thermo Fisher Scientific, Waltham, Massachusetts, United States). Appropriate control procedures were used to guarantee detection accuracy; further details are provided in our previous study (Zhao et al., 2020).

### 2.3 Covariates

Trained undergraduate and postgraduate students interviewed each participant to determine their age (continuous variable), sex (male or female), smoking status (yes or no), and body mass index (BMI; calculated using measured height and weight). Urine creatinine (U-Cre) levels were measured by staff members at a local tertiary hospital to avoid urine dilution. Because methylation has cell-type specificity and blood samples comprise various cell types, CpG methylation levels and methylation array data can be confounded by cell-type composition (Jaffe and Irizarry, 2014). To estimate cell-type composition in blood samples, we used the Houseman regression calibration method (Houseman et al., 2012) in the minfi package to evaluate the proportions of CD8^+^ T cells, CD4^+^ T cells, natural killer (NK) cells, B cells, monocytes, and granulocytes in each blood sample.

### 2.4 DNA methylation analysis

Fasting whole blood samples were collected by clinicians in ethylenediaminetetraacetic acid (EDTA) anticoagulant tubes and stored them at −80°C. A DNeasy Blood and Tissue Kit (Qiagen) was used to isolate DNA from whole blood. The purity and concentration of DNA were estimated using Nanodrop 2000 (Thermo Fisher Scietific, Waltham, Massachusetts, United States). Approximately 500 ng of genomic DNA from each sample was used for sodium bisulfite conversion with the EZ DNA methylation Gold Kit (Zymo Research, United States) in accordance with the manufacturer’s standard protocol. Genome-wide DNA methylation was performed using the Illumina Infinium HumanMethylation850K (EPIC) BeadChip (Illumina Inc, United States) in accordance with the manufacturer’s instructions.

### 2.5 Data processing

The original array data (IDAT files) were preprocessed using the R (4.1.2) Bioconductor package (ChAMP) for deriving DNA methylation levels. We excluded unhybridized probes (detection *p* value >0.01) and probes with a maximum of two beads in 5% of samples. Non-CpG probes, single-nucleotide polymorphisms (SNPs) related probes, and all multi-hit probes were excluded. Probes mapping to sex chromosomes (X and Y) were also excluded to avoid sex-specific methylation bias (Zeng et al., 2019). In total, 797,055 high-quality probes remained for subsequent analysis. We performed probe-type normalization by using the beta-mixture quantile method (Marabita et al., 2013). The methylation status of all CpG sites was denoted as a beta value between 0 (completely unmethylated) and 1 (completely methylated) after preprocessing, involving both control normalization and background subtraction. Finally, we used the champ. runCombat function in the ChAMP package to adjust for batch effects, and principal component analysis was performed to determine the effectiveness of batch effect removal (Wu et al., 2017).

### 2.6 Data analysis

Medians (interquartile ranges, IQRs) and frequencies (proportions) were used as continuous and categorical variables respectively, to present the descriptive statistics of the participant’s demographics and Cr concentrations. We examined differences between groups with and without Cr exposure by using the Mann–Whitney *U* test for continuous variables and Fisher’s exact test for categorical variables. We also evaluated the associations between log_2_ (urinary Cr) concentrations and estimated whole blood cell-type proportions (CD8^+^ T cells, CD4^+^ T cells, NK cells, B cells, monocytes, and granulocytes) by using linear models adjusted for sex, age, smoking status, and BMI.

Prior to further analysis, log_2_ transformation was performed on Cr concentrations to eliminate the effects of extreme values because the distribution was right skewed. We conducted an epigenome-wide association study (EWAS) for investigating DMPs through linear regression in the limma package in R with empirical Bayes smoothing of standard errors (Ritchie et al., 2015). Beta values were logit-transformed to M values $\ln\left[Beta\;value/\left(1-Beta\;value\right)\right]$ to better meet model assumptions (Du et al., 2010). A robust linear regression model was constructed with M values as dependent variables and log_2_ transformed Cr concentration as the independent variable after adjustment for sex, smoking status, BMI, urine creatinine level, age at enrollment, and estimated cell-type proportions. With consideration for multiple comparisons, the Benjamini and Hochberg (1995) method for false discovery rates (FDRs) was applied using the p. adjust function in R, and Bonferroni correction values were calculated as *p* values multiplied by the number of tests (Bozack et al., 2020), with significance set to α = 0.05. Because the biological interpretation of beta values is straightforward (Du et al., 2010), the effect estimates are reported as beta values with 95% confidence intervals. In sensitivity analysis, the exposed/unexposed group was also used as an independent variable adjusting the same covariates to confirm the robustness.

We analyzed DMRs using DMRcate (Peters et al., 2015) at a bandwidth of 1,000 nucleotides ( λ = 1,000), and the scaling factor for bandwidth was C = 2 (recommended parameter for EPIC arrays). DMRcate identifies the most differentially methylated regions by agglomerating CpG locations with an adjusted *p* value below a certain threshold (based on FDRs), and these regions’ locations are at post-smoothed significant probes where the distance to the next consecutive probe is less than λ (1,000) nucleotides. All data analysis was conducted using R version 4.1.2.

## 3 Results

### 3.1 Participant characteristics

Table 1 presents the demographic characteristics of the participants. The groups did not differ significantly in terms of sex, age, smoking status, or BMI distribution (*p* > 0.05). The median urine Cr concentration in exposure group was 9.4 μg/L, significantly higher than that in the control group (3.5 μg/L, *p* < 0.001). In the exposure group, the median U-Cre level was 120.0 mg/dl, significantly higher than that in the unexposed group (53.8 mg/dl, *p* < 0.001). In the linear models adjusted for sex, smoking status, BMI, urine creatinine concentration, and age at enrollment, Cr exposure was not associated with all cell types (CD8^+^ T cells, CD4^+^ T cells, NK cells, B cells, monocytes, and granulocytes), as estimated using Houseman regression calibration (Supplementary Table S1).

**TABLE 1:** Characteristics of chromate exposed and unexposed groups.

	Exposed group (*n* = 10)		Unexposed group (*n* = 10)		*p*-Value
	N or median	% or IQR	N or median	% or IQR	
Age (year)	65.5	(62.5, 68.5)	64.0	(56.5, 66.0)	0.449
BMI (kg/m^2^)	24.0	(22.3, 27.0)	24.5	(22.7, 25.7)	0.971
Sex					>0.999
male	5	50%	4	40%	
female	5	50%	6	60%	
Smoker					>0.999
yes	4	40%	5	50%	
no	6	60%	5	50%	
Urine Cr (μg/L)	9.4	(5.5, 18.9)	3.5	(2.8, 3.7)	<0.001
Urine creatinine (mg/dl)	120.0	(84.1,233.1)	53.8	(39.5, 76.9)	0.009

Fisher’s exact test was used for categorical variables. A non-parametric test (Mann–Whitney *U* test) was performed for comparisons between groups IQR, interquartile range.

### 3.2 Differentially methylated positions

**FIGURE 1:**
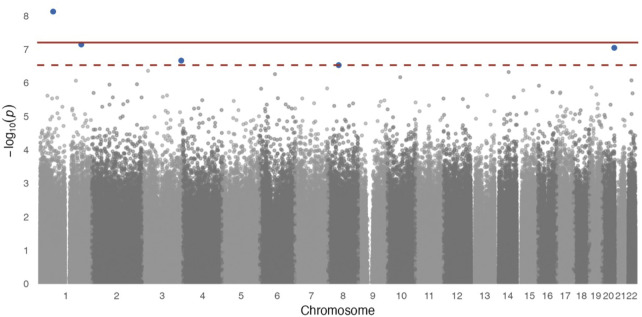
Manhattan plot for epigenome-wide association of log_2_ transformed urinary Cr concentration and DNA methylation levels. Full models were adjusted for sex, smoking status, BMI, urine creatinine level, age at enrollment, and estimated cell-type proportions. The solid red line represents the Bonferroni cutoff, and the dashed line represents the FDR cutoff.

**FIGURE 2:**
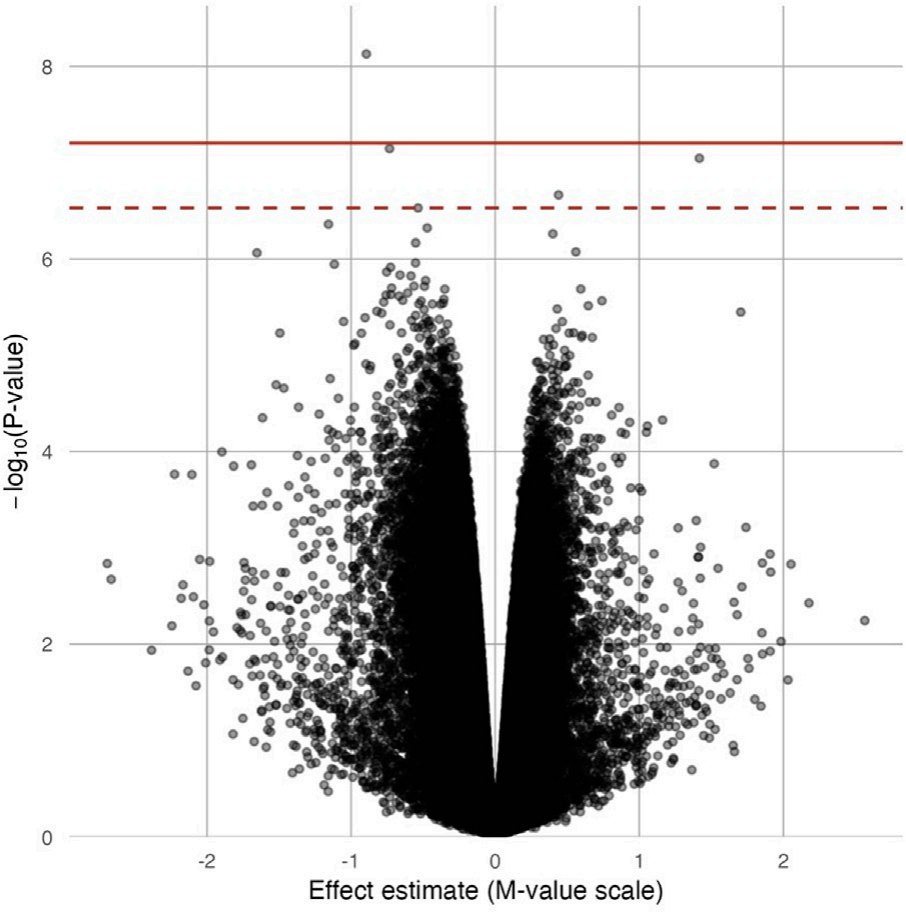
Volcano plot for the epigenome-wide association of log_2_ transformed urinary Cr concentration and DNA methylation levels. Full models were adjusted for sex, smoking status, BMI, urine creatinine level, age at enrollment, and estimated cell-type proportions. The solid red line represents the Bonferroni cutoff, and the dashed line represents the FDR cutoff.

In site-specific analyses, 797,055 methylated CpG sites were tested in fully adjusted models with log_2_ (urinary Cr) values; Figure 1 presents the Manhattan plot. After multiple comparisons with FDRs and the Bonferroni correction, five (*p*_FDR_ < 0.05) DMPs (annotations relevant to four genes) were significantly associated with Cr exposure [cg20690919 (*USP1*), cg00704664 (*CDH4*), cg10809143 (*IL1RAP*), cg27057652 (*PXDNL*), and cg05390480], and only cg20690919 remained significant at a Bonferroni *p* value of <0.05 (Table 2). Each two-fold increase in Cr concentration in urine was associated with an 11.03% greater methylation of cg00704664 (95% CI: 8.34, 13.73; *p* = 9.02 × 10^−8^, *p*_FDR_ = 2.40 × 10^−2^) and a 3.22% increase in the methylation of cg10809143 (95% CI: 2.52, 3.92; *p* = 2.17 × 10^−7^, *p*_FDR_ = 4.32 × 10^−2^; Table 2). At three DMPs (cg20690919: β = −2.80; cg05390480: β = −1.58; cg27057652: β = −3.16), decreased methylation was associated with Cr concentration. In Figure 2, most of the methylated CpG sites were hypomethylation: 60.9% of probes were hypomethylation while 39.1% of all probes were hypermethylation. In the sensitivity analysis using exposure group, the results were similar (Supplementary Table S2).

**TABLE 2:** Differentially methylated CpG sites associated with Cr exposure (*p*_FDR_ < 0.05).

CpG	Chromosome	Position^a^	Gene	*p*-Value	*p*_FDR_	*p*_Bonferroni_	Median methylation (%) of unexposed group^b^	Median methylation (%) of exposed group^b^	Mean difference in % methylation (95%CI)^c^
cg20690919	1	62901654	USP1	7.44 × 10^−9^	0.006	0.006	9.8	1.8	−2.80 (−3.64, −1.96)
cg05390480	1	199059142	—	7.16 × 10^−8^	0.024	0.057	6.8	1.6	−1.58 (−2.27, −0.89)
cg00704664	20	60500578	CDH4	9.02 × 10^−8^	0.024	0.072	2.3	18.1	11.03 (8.34, 13.73)
cg10809143	3	190281145	IL1RAP	2.17 × 10^−7^	0.043	0.173	83.5	91.8	3.22 (2.52, 3.92)
cg27057652	8	52322137	PXDNL	2.96 × 10^−7^	0.047	0.236	95.2	86.4	−3.16 (−3.99, −2.33)

^a^ hg19 assembly.

^b^ Calculated beta value × 100.

^c^ Effect size estimate from limma models of beta values adjusted for sex, smoking status, BMI, urine creatinine level, age at enrollment, and estimated cell-type proportions.

### 3.3 Differentially methylated regions

**FIGURE 3:**
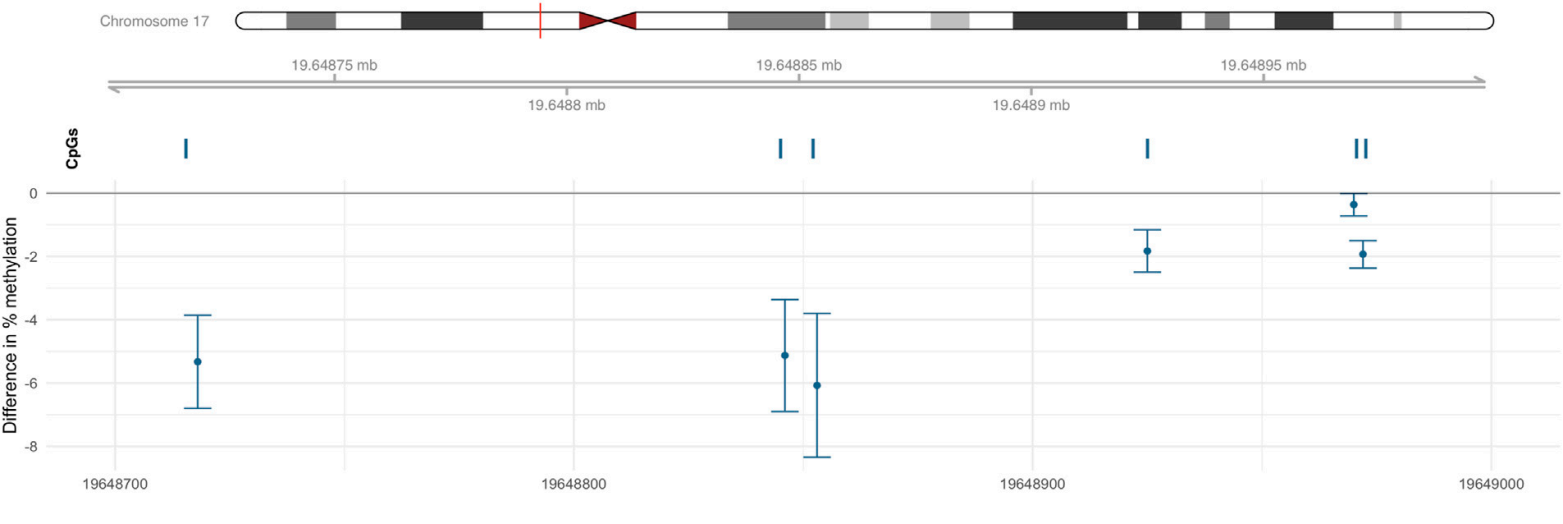
DMR associated with Cr located within chr17: 19,648,718–19,648,972. Percentage difference in methylation estimated from limma models of beta values adjusted for sex, smoking status, BMI, urine creatinine level, age at enrollment, and estimated cell-type proportions.

One DMR (chr17: 19,648,718–19,648,972) was identified on chromosome 17 as containing 6 CpGs (Figure 3); this six CpGs are ranked by genomic position in Table 3. This DMR spans the body, the first exon, TSS200, TSS1500, and the 5′UTR of *ALDH3A1*. The DNA methylation levels in this region were high, ranging from 64.90% to 94.66%. All six CpG sites in this region were significantly and negatively associated with Cr level at nominal *p* < 0.05.

**TABLE 3:** CpG sites located in DMR (chr17: 19,648,718–19,648,972).

CpG	Position^a^	Gene	Feature category	*p*-Value	pFDR	Median methylation (%) of unexposed group^b^	Median methylation (%) of exposed group^b^	Mean difference in % methylation (95%CI)^c^
cg15046965	19648718	ALDH3A1	5′UTR; 1stExon	2.22 × 10^−5^	0.072	73.0	55.2	−5.33 (−6.80, −3.86)
cg23098051	19648846	ALDH3A1	1stExon; 5′UTR; TSS200	1.20 × 10^−4^	0.083	91.2	76.1	−5.13 (−6.90, −3.36)
cg11475454	19648853	ALDH3A1	1stExon; 5′UTR; TSS200	2.19 × 10^−4^	0.089	77.7	58.7	−6.08 (−8.35, −3.80)
cg05447343	19648925	ALDH3A1	5′UTR; TSS200	1.77 × 10^−4^	0.087	92.0	82.6	−1.83 (−2.49, −1.16)
cg18957070	19648970	ALDH3A1	5′UTR; TSS200	4.70 × 10^−2^	0.320	95.5	93.5	−0.36 (−0.72, −0.01)
cg01471036	19648972	ALDH3A1	5′UTR; TSS1500; TSS200	4.10 × 10^−6^	0.070	93.6	88.1	−1.93 (−2.37, −1.50)

^a^ hg19 assembly.

^b^ Calculated beta value × 100.

^c^ Effect size estimated from limma models of beta values adjusted for sex, smoking status, BMI, urine creatinine, age at enrollment, and estimated cell-type proportions.

## 4 Discussion

Although the carcinogenic mechanism of Cr exposure is unclear, genomic instability and epigenetic modification have been identified during molecular processes linked to cancer (Browning et al., 2017; Rager et al., 2019). In addition, *in vivo* Cr-related data illustrated genotoxicity and mutagenicity results have mostly been negative, and the mutagenic is not the main cause of Cr (VI)-induced cancer (Proctor et al., 2014; Browning et al., 2017). Therefore, epigenetic modification, especially DNA methylation, may play a vital role in pathogenesis mechanism of Cr (VI) (Feng et al., 2020). Our EWAS identified an association of moderate to high levels of Cr exposure with whole-blood DNA methylation, and we identified five CpGs annotated to four genes at *p*_FDR_ < 0.05: cg20690919 (*USP1*), cg00704664 (*CDH4*), cg10809143 (*IL1RAP*), cg27057652 (*PXDNL*), and cg05390480. We also identified one DMR (chr17: 19,648,718–19,648,972).

The most significant Cr-related CpG, cg20690919, is located in the gene *ubiquitin specific peptidase 1* (*USP1*) gene, which is a 785 amino-acid deubiquitinating enzyme with His and Cys domains (Jang and Kim, 2021). *USP1* can involve in DNA interstrand crosslink repair by deubiquitinating the Fanconi anemia protein (Taniguchi et al., 2002; Kim and D'Andrea, 2012) and regulating DNA damage response pathways (Jang and Kim, 2021). USP1-associated factor 1 (*UAF1*) and *RAD51*-associated protein 1 (*RAD51AP1*) enhance the activity of recombinase *RAD51* during DNA repair through the homologous recombination pathway (Liang et al., 2020). *RAD51* has been significantly downregulated in response to Cr exposure in cell studies (Clancy et al., 2012; Wu et al., 2012), and in a study related to occupational Cr exposure, *RAD51* was modified by hypermethylation to promote gene damage (Hu et al., 2018).

Additional DMPs may be biologically responsive to Cr exposure. The *CDH4* gene encodes retinal cadherin (R-cadherin), and the deregulation of R-cadherin is implicated in several human cancers (Miotto et al., 2004; Agiostratidou et al., 2009). *CDH4* is epigenetically silenced through promoter hypermethylation in some epithelial cancers, and it may act as a tumor suppressor (Tang et al., 2018). *IL1RAP* encodes a component of the interleukin 1 receptor complex which initiates signaling events that result in the activation of interleukin 1–responsive genes. Adam et al. indicated that Cr (VI)-induced cutaneous toxicity causes mitochondrial reactive oxygen species accumulation, resulting in increased IL-1β processing and secretion (Adam et al., 2017). However, few studies have investigated the biological function of PXDNL, and we did not find evidence confirming the relationship between *PXDNL* methylation level and Cr exposure.

Several studies have revealed significant associations between Cr exposure and stomach cancer, and a meta-analysis of 56 cohort and case–control studies suggested that Cr (VI) is a stomach carcinogen in humans (relative risk = 1.27, *95% CI:* 1.18–1.38) (Welling et al., 2015). Additionally, several potential mechanisms of heavy metal (Cr included) exposure contribute to GC development (Fink, 2003; Chervona et al., 2012; Yuan et al., 2016), and in our previous work, we observed that exposure to Cr in the environment leads to chronic digestive system damage (Meng et al., 2015). In this study, three of these four differentially methylated genes have been reported to be associated with digestive system cancers (*USP1*, *CDH4*, and *IL1RAP*). A study involving 188 patients with gastric cancer (GC) revealed that the mRNA levels and protein levels of *USP1* were overexpressed in GC tissue than in adjacent normal tissue, and further analysis indicated that USP1 may promote GC metastasis by upregulating ID2 expression (Li et al., 2021). Meng et al. revealed that USP1 was upregulated in GC tissue and played an oncogenic role in GC progression (Meng and Li, 2022). An analysis of human primary tumors indicated that *CDH4* was hypermethylated in colorectal (78%) and gastric (95%) carcinomas, and *CDH4* methylation may be an early event in gastrointestinal tumor progression (Miotto et al., 2004). A bioinformatics analysis identified differentially methylated *CDH4* in human colon cancer samples (Luo et al., 2021). *In vitro* and *in vivo* data have indicated that *IL1RAP* knockdown significantly increased inflammation of tumor microenvironment-related inflammatory factors and suppressed the development of stomach carcinoma (Lv et al., 2021). Therefore, the results of our study suggest that Cr exposure might cause gastrointestinal diseases through DNA methylation and related pathways. As no epigenomic study has explored the relationship between Cr exposure and gastrointestinal disease at DNA methylation levels, Cr-related studies focusing on gastrointestinal diseases are needed to further confirm it.

Our study identified one DMR (chr17: 19,648,718–19,648,972) that spanned several functional regions (including body, the first exon, TSS200, TSS1500, and 5′UTR) of aldehyde dehydrogenase 3A1 (*ALDH3A1*). *ALDH3A1* is an NAD (P)^+^-dependent enzyme with the capacity to oxidize medium-chain aliphatic and aromatic aldehydes (Marchitti et al., 2008; Voulgaridou et al., 2017). Although no study has reported an association between *ALDH3A1* and metal exposure, through the inhibition (Muzio et al., 2001; Oraldi et al., 2011) or activation (Leong et al., 2003; Vitale-Brovarone et al., 2008) of *ALDH3A1* pathways, studies have demonstrated the close relationship between *ALDH3A1* and cell proliferation. Meanwhile, a study exploring the gene expression and DNA methylation changes in current and ex-smokers found that lower methylation CpG sites of ALDH*3A1* increased the expression levels of *ALDH3A1* (Reddy et al., 2021). This may play a key role in tumor promotion because elevated expression levels of *ALDH3A1* were linked to increased cell growth and survival (Burchiel et al., 2007).

Studies have demonstrated the role of epigenetic alterations in Cr (VI)-induced carcinogenicity and indicated that epigenetic alterations lead to tumorigenesis through cell proliferation (Pillai et al., 2011; Clancy et al., 2012; Teng et al., 2013; Nguewa et al., 2014), DNA damage (Xie et al., 2005), and DNA repair (Suter et al., 2004; Baylin, 2005). One review proposed that Cr (VI)-induced decreases in DNA repair signaling concomitant with increased cell proliferation increased the risk of DNA damage through cell replication and that such damage could lead to genomic instability and carcinogenesis (Rager et al., 2019). We identified significant DMPs and DMRs related to DNA repair and cell proliferation.

Most epidemiological studies have focused on the relationship between occupational exposure to Cr and DNA methylation (Kondo et al., 2006; Hu et al., 2018; Feng et al., 2020; Ha et al., 2021). However, previous studies only focused on occupational population but were not generalizable to general population environmental-exposed to Cr (VI). The median urinary Cr concentration of Chinese individuals in a national survey was 0.38 (IQR: 0.15, 0.72) μg/L (Cao et al., 2021). The median urinary Cr concentrations of participants enrolled in this study were 9.4 μg/L and 3.5 μg/L, which are higher than the median level of Chinese individuals. This study focused on populations with relatively high levels of Cr exposure and provided epidemiological evidence of the effects of high levels of Cr exposure on DNA methylation. To the best of our knowledge, this is the first study to discuss the association of environmental Cr exposure and DNA methylation and explore high levels of Cr-induced epigenetic effects. Another major strength of our study is the use of EPIC microarray data to detect DNA methylation. DNA methylation in other EWASs (Hu et al., 2018; Zeng et al., 2019; Feng et al., 2020) on Cr exposure has been measured through 450K microarray, which only interrogates about 480,000 CpGs; EPIC microarray interrogates >850,000 CpGs, comprising >90% of 450K CpGs. By using EPIC microarray, our study enlarges the DNA methylation sites and increases power in EWAS through the correction for multiple testing (Bozack et al., 2020).

This study has some limitations that should be considered. First, although this study identified five significant DMP- and DMR-related genes (*CDH4*, *IL1RAP*, *USP1*, *PXDNL*, and *ALDH3A1*), we did not examine the mRNA expression and translation levels of these genes. Thus, we could not determine whether Cr exposure affected the function of these differential genes. Second, statistical power was limited by the study’s small sample; thus, we may not have accurately identified differences in methylation between the exposed and unexposed groups, although we performed more comparisons than other studies and our standard for the comparisons were strict. As this study has been performed in a limited number of individuals, we acknowledged that this study was preliminary and research involving larger samples is needed to confirm our findings. Third, some unconsidered confounding factors could bias our results. However, the similar lifestyles, cultural backgrounds, and socioeconomic status of the participants; the high-quality control procedures; and the Houseman regression calibration method, which accounts for different cell-type compositions, can reduce bias. The epigenetic effect of Cr should be further explored in different cell types because DNA methylation might be specific to certain cell types (Lu et al., 2021).

## 5 Conclusion

This is the first study to explore the relationship between environmental Cr exposure and epigenome-wide DNA methylation and to use 850K microarray to investigate Cr (VI)-induced epigenetic alterations. In a cross-sectional study focusing on Jinzhou City, which has high levels of Cr exposure, five significant CpGs that annotated four genes were identified at *p*_FDR_ < 0.05. In addition, a DMR-annotated gene (*ALDH3A1*) related to cell proliferation and tumor promotion was identified. Epidemiological and biological studies should use larger samples to provide evidence to support out findings regarding the epigenetic effects of environmental Cr exposure.

## Data Availability

The data presented in the study are deposited in the GEO repository, accession number GSE206418, https://www.ncbi.nlm.nih.gov/geo/query/acc.cgi?acc=GSE206418.

## References

[B1] Abreu P. L. Cunha-Oliveira T. Ferreira L. M. R. Urbano A. M. (2018). Hexavalent chromium, a lung carcinogen, confers resistance to thermal stress and interferes with heat shock protein expression in human bronchial epithelial cells. Biometals 31 (4), 477–487. 10.1007/s10534-018-0093-7 29549560

[B2] Adam C. Wohlfarth J. Haußmann M. Sennefelder H. Rodin A. Maler M. (2017). Allergy-inducing chromium compounds trigger potent innate immune stimulation via ROS-dependent inflammasome activation. J. Invest. Dermatol 137 (2), 367–376. 10.1016/j.jid.2016.10.003 27751866

[B3] Agiostratidou G. Li M. Suyama K. Badano I. Keren R. Chung S. (2009). Loss of retinal cadherin facilitates mammary tumor progression and metastasis. Cancer Res. 69 (12), 5030–5038. 10.1158/0008-5472.CAN-08-4007 19491271 PMC4382672

[B4] Baylin S. B. (2005). DNA methylation and gene silencing in cancer. Nat. Clin. Pract. Oncol. 2, S4–S11. 10.1038/ncponc0354 16341240

[B5] Benjamini Y. Hochberg Y. (1995). Controlling the false discovery rate: A practical and powerful approach to multiple testing. J. R. Stat. Soc. Ser. B Methodol. 57 (1), 289–300. 10.1111/j.2517-6161.1995.tb02031.x

[B6] Bozack A. K. Domingo-Relloso A. Haack K. Gamble M. V. Tellez-Plaza M. Umans J. G. (2020). Locus-specific differential DNA methylation and urinary arsenic: An epigenome-wide association study in blood among adults with low-to-moderate arsenic exposure. Environ. Health Perspect. 128 (6), 67015. 10.1289/EHP6263 32603190 PMC7534587

[B7] Brocato J. Costa M. (2013). Basic mechanics of DNA methylation and the unique landscape of the DNA methylome in metal-induced carcinogenesis. Crit. Rev. Toxicol. 43 (6), 493–514. 10.3109/10408444.2013.794769 23844698 PMC3871623

[B8] Browning C. L. Speer R. M. Wise J. P. (2017). “Molecular mechanisms of chromium-induced carcinogenesis,” in Essential and non-essential metals: Carcinogenesis, prevention and cancer therapeutics. Editors Mudipalli A. Zelikoff J. T. (Cham: Springer International Publishing), 143–180.

[B9] Burchiel S. W. Thompson T. A. Lauer F. T. Oprea T. I. (2007). Activation of dioxin response element (DRE)-associated genes by benzo(a)pyrene 3, 6-quinone and benzo(a)pyrene 1, 6-quinone in MCF-10A human mammary epithelial cells. Toxicol. Appl. Pharmacol. 221 (2), 203–214. 10.1016/j.taap.2007.02.020 17466351 PMC2020824

[B10] Cantone I. Fisher A. G. (2013). Epigenetic programming and reprogramming during development. Nat. Struct. Mol. Biol. 20 (3), 282–289. 10.1038/nsmb.2489 23463313

[B11] Cao Z. Lin S. Zhao F. Lv Y. Qu Y. Hu X. (2021). Cohort profile: China national human biomonitoring (CNHBM)-A nationally representative, prospective cohort in Chinese population. Environ. Int. 146, 106252. 10.1016/j.envint.2020.106252 33242729 PMC7828642

[B12] Chervona Y. Arita A. Costa M. (2012). Carcinogenic metals and the epigenome: Understanding the effect of nickel, arsenic, and chromium. Metallomics 4 (7), 619–627. 10.1039/c2mt20033c 22473328 PMC3687545

[B13] Clancy H. A. Sun H. Passantino L. Kluz T. Muñoz A. Zavadil J. (2012). Gene expression changes in human lung cells exposed to arsenic, chromium, nickel or vanadium indicate the first steps in cancer. Metallomics 4 (8), 784–793. 10.1039/c2mt20074k 22714537 PMC3563094

[B14] Du P. Zhang X. Huang C. C. Jafari N. Kibbe W. A. Hou L. (2010). Comparison of Beta-value and M-value methods for quantifying methylation levels by microarray analysis. BMC Bioinforma. 11, 587. 10.1186/1471-2105-11-587 PMC3012676 21118553

[B15] Dworzański W. Cholewińska E. Fotschki B. Juśkiewicz J. Listos P. Ognik K. (2020). Assessment of DNA methylation and oxidative changes in the heart and brain of rats receiving a high-fat diet supplemented with various forms of chromium. Anim. (Basel) 10 (9), 1470. 10.3390/ani10091470 PMC7552180 32825649

[B16] Feng L. Guo X. Li T. Yao C. Xia H. Jiang Z. (2020). Novel DNA methylation biomarkers for hexavalent chromium exposure: An epigenome-wide analysis. Epigenomics 12 (3), 221–233. 10.2217/epi-2019-0216 31961222

[B17] Fink M. P. (2003). Intestinal epithelial hyperpermeability: Update on the pathogenesis of gut mucosal barrier dysfunction in critical illness. Curr. Opin. Crit. Care 9 (2), 143–151. 10.1097/00075198-200304000-00011 12657978

[B18] Ha F. Li N. Long C. Zheng P. Hu G. Jia G. (2021). The effect of global DNA methylation on PDCD5 expression in the PBMC of occupational chromate exposed workers. J. Occup. Environ. Med. 63 (7), 600–608. 10.1097/JOM.0000000000002192 34184653

[B19] Houseman E. A. Accomando W. P. Koestler D. C. Christensen B. C. Marsit C. J. Nelson H. H. (2012). DNA methylation arrays as surrogate measures of cell mixture distribution. BMC Bioinforma. 13, 86. 10.1186/1471-2105-13-86 PMC3532182 22568884

[B20] Hu G. Li P. Cui X. Li Y. Zhang J. Zhai X. (2018). Cr(VI)-induced methylation and down-regulation of DNA repair genes and its association with markers of genetic damage in workers and 16HBE cells. Environ. Pollut. 238, 833–843. 10.1016/j.envpol.2018.03.046 29627753

[B21] Jaffe A. E. Irizarry R. A. (2014). Accounting for cellular heterogeneity is critical in epigenome-wide association studies. Genome Biol. 15 (2), R31. 10.1186/gb-2014-15-2-r31 24495553 PMC4053810

[B22] Jang S. W. Kim J. M. (2021). Mutation of aspartic acid 199 in USP1 disrupts its deubiquitinating activity and impairs DNA repair. FEBS Lett. 595 (15), 1997–2006. 10.1002/1873-3468.14152 34128540

[B23] Kim H. D'Andrea A. D. (2012). Regulation of DNA cross-link repair by the Fanconi anemia/BRCA pathway. Genes Dev. 26 (13), 1393–1408. 10.1101/gad.195248.112 22751496 PMC3403008

[B24] Kondo K. Takahashi Y. Hirose Y. Nagao T. Tsuyuguchi M. Hashimoto M. (2006). The reduced expression and aberrant methylation of p16(INK4a) in chromate workers with lung cancer. Lung Cancer 53 (3), 295–302. 10.1016/j.lungcan.2006.05.022 16828922

[B25] Kothari C. Ouellette G. Labrie Y. Jacob S. Diorio C. Durocher F. (2018). Identification of a gene signature for different stages of breast cancer development that could be used for early diagnosis and specific therapy. Oncotarget 9 (100), 37407–37420. 10.18632/oncotarget.26448 30647841 PMC6324778

[B26] Laird P. W. (2003). The power and the promise of DNA methylation markers. Nat. Rev. Cancer 3 (4), 253–266. 10.1038/nrc1045 12671664

[B27] Leong K. F. Cheah C. M. Chua C. K. (2003). Solid freeform fabrication of three-dimensional scaffolds for engineering replacement tissues and organs. Biomaterials 24 (13), 2363–2378. 10.1016/s0142-9612(03)00030-9 12699674

[B28] Li N. Wu L. Zuo X. Luo H. Sheng Y. Yan J. (2021). USP1 promotes GC metastasis via stabilizing ID2. Dis. Markers 2021, 3771990. 10.1155/2021/3771990 34873426 PMC8643267

[B29] Liang F. Miller A. S. Tang C. Maranon D. Williamson E. A. Hromas R. (2020). The DNA-binding activity of USP1-associated factor 1 is required for efficient RAD51-mediated homologous DNA pairing and homology-directed DNA repair. J. Biol. Chem. 295 (24), 8186–8194. 10.1074/jbc.RA120.013714 32350107 PMC7294083

[B30] Lou J. Wang Y. Yao C. Jin L. Wang X. Xiao Y. (2013). Role of DNA methylation in cell cycle arrest induced by Cr (VI) in two cell lines. PLoS One 8 (8), e71031. 10.1371/journal.pone.0071031 23940686 PMC3735518

[B31] Lu T. Cardenas A. Perron P. Hivert M. F. Bouchard L. Greenwood C. M. T. (2021). Detecting cord blood cell type-specific epigenetic associations with gestational diabetes mellitus and early childhood growth. Clin. Epigenetics 13 (1), 131. 10.1186/s13148-021-01114-5 34174944 PMC8236204

[B32] Luo Y. Sun F. Peng X. Dong D. Ou W. Xie Y. (2021). Integrated bioinformatics analysis to identify abnormal methylated differentially expressed genes for predicting prognosis of human colon cancer. Int. J. Gen. Med. 14, 4745–4756. 10.2147/IJGM.S324483 34466019 PMC8403012

[B33] Lv Q. Xia Q. Li A. Wang Z. (2021). The potential role of IL1RAP on tumor microenvironment-related inflammatory factors in stomach adenocarcinoma. Technol. Cancer Res. Treat. 20, 1533033821995282. 10.1177/1533033821995282 33602046 PMC7897808

[B34] Marabita F. Almgren M. Lindholm M. E. Ruhrmann S. Fagerström-Billai F. Jagodic M. (2013). An evaluation of analysis pipelines for DNA methylation profiling using the Illumina HumanMethylation450 BeadChip platform. Epigenetics 8 (3), 333–346. 10.4161/epi.24008 23422812 PMC3669124

[B35] Marchitti S. A. Brocker C. Stagos D. Vasiliou V. (2008). Non-P450 aldehyde oxidizing enzymes: The aldehyde dehydrogenase superfamily. Expert Opin. Drug Metab. Toxicol. 4 (6), 697–720. 10.1517/17425255.4.6.697 18611112 PMC2658643

[B36] Marino N. German R. Podicheti R. Rusch D. B. Rockey P. Huang J. (2022). Aberrant epigenetic and transcriptional events associated with breast cancer risk. Clin. Epigenetics 14 (1), 21. 10.1186/s13148-022-01239-1 35139887 PMC8830042

[B37] Martinez-Zamudio R. Ha H. C. (2011). Environmental epigenetics in metal exposure. Epigenetics 6 (7), 820–827. 10.4161/epi.6.7.16250 21610324 PMC3230540

[B38] Meng D. Li D. (2022). Ubiquitin-specific protease 1 overexpression indicates poor prognosis and promotes proliferation, migration, and invasion of gastric cancer cells. Tissue Cell 74, 101723. 10.1016/j.tice.2021.101723 34990966

[B39] Meng W. Liu X. Wei L. Xiao C. Xu Q. (2015). Investigation of digestive system damage of residents in areas polluted by hexavalent chromium. Basic & Clin. Med. 35 (06), 772–775.

[B40] Miotto E. Sabbioni S. Veronese A. Calin G. A. Gullini S. Liboni A. (2004). Frequent aberrant methylation of the CDH4 gene promoter in human colorectal and gastric cancer. Cancer Res. 64 (22), 8156–8159. 10.1158/0008-5472.CAN-04-3000 15548679

[B41] Muzio G. Canuto R. A. Trombetta A. Maggiora M. (2001). Inhibition of cytosolic class 3 aldehyde dehydrogenase by antisense oligonucleotides in rat hepatoma cells. Chem. Biol. Interact. 130-132 (1-3), 219–225. 10.1016/s0009-2797(00)00281-7 11306046

[B42] Nguewa P. Manrique I. Díaz R. Redrado M. Parrondo R. Perez-Stable C. (2014). Id-1B, an alternatively spliced isoform of the inhibitor of differentiation-1, impairs cancer cell malignancy through inhibition of proliferation and angiogenesis. Curr. Mol. Med. 14 (1), 151–162. 10.2174/1566524013666131203100643 24295493

[B43] Oraldi M. Saracino S. Maggiora M. Chiaravalloti A. Buemi C. Martinasso G. (2011). Importance of inverse correlation between ALDH3A1 and PPARγ in tumor cells and tissue regeneration. Chem. Biol. Interact. 191 (1-3), 171–176. 10.1016/j.cbi.2011.01.011 21251908

[B44] Peters T. J. Buckley M. J. Statham A. L. Pidsley R. Samaras K. Reginald V. L. (2015). De novo identification of differentially methylated regions in the human genome. Epigenetics Chromatin 8, 6. 10.1186/1756-8935-8-6 25972926 PMC4429355

[B45] Pillai S. Rizwani W. Li X. Rawal B. Nair S. Schell M. J. (2011). ID1 facilitates the growth and metastasis of non-small cell lung cancer in response to nicotinic acetylcholine receptor and epidermal growth factor receptor signaling. Mol. Cell Biol. 31 (14), 3052–3067. 10.1128/MCB.01311-10 21606196 PMC3133413

[B46] Proctor D. M. Suh M. Campleman S. L. Thompson C. M. (2014). Assessment of the mode of action for hexavalent chromium-induced lung cancer following inhalation exposures. Toxicology 325, 160–179. 10.1016/j.tox.2014.08.009 25174529

[B47] Rager J. E. Suh M. Chappell G. A. Thompson C. M. Proctor D. M. (2019). Review of transcriptomic responses to hexavalent chromium exposure in lung cells supports a role of epigenetic mediators in carcinogenesis. Toxicol. Lett. 305, 40–50. 10.1016/j.toxlet.2019.01.011 30690063

[B48] Reddy K. D. Lan A. Boudewijn I. M. Rathnayake S. N. H. Koppelman G. H. Aliee H. (2021). Current smoking alters gene expression and DNA methylation in the nasal epithelium of patients with asthma. Am. J. Respir. Cell Mol. Biol. 65 (4), 366–377. 10.1165/rcmb.2020-0553OC 33989148

[B49] Ritchie M. E. Phipson B. Wu D. Hu Y. Law C. W. Shi W. (2015). Limma powers differential expression analyses for RNA-sequencing and microarray studies. Nucleic Acids Res. 43 (7), e47. 10.1093/nar/gkv007 25605792 PMC4402510

[B50] Santonen T. Alimonti A. Bocca B. Duca R. C. Galea K. S. Godderis L. (2019). Setting up a collaborative European human biological monitoring study on occupational exposure to hexavalent chromium. Environ. Res. 177, 108583. 10.1016/j.envres.2019.108583 31330491

[B51] Suter C. M. Martin D. I. Ward R. L. (2004). Germline epimutation of MLH1 in individuals with multiple cancers. Nat. Genet. 36 (5), 497–501. 10.1038/ng1342 15064764

[B52] Tang Q. Lu J. Zou C. Shao Y. Chen Y. Narala S. (2018). CDH4 is a novel determinant of osteosarcoma tumorigenesis and metastasis. Oncogene 37 (27), 3617–3630. 10.1038/s41388-018-0231-2 29610525

[B53] Taniguchi T. Garcia-Higuera I. Xu B. Andreassen P. R. Gregory R. C. Kim S. T. (2002). Convergence of the fanconi anemia and ataxia telangiectasia signaling pathways. Cell 109 (4), 459–472. 10.1016/s0092-8674(02)00747-x 12086603

[B54] Teng Y. C. Lee C. F. Li Y. S. Chen Y. R. Hsiao P. W. Chan M. Y. (2013). Histone demethylase RBP2 promotes lung tumorigenesis and cancer metastasis. Cancer Res. 73 (15), 4711–4721. 10.1158/0008-5472.CAN-12-3165 23722541

[B55] Tiffon C. (2018). The impact of nutrition and environmental epigenetics on human health and disease. Int. J. Mol. Sci. 19 (11), 3425. 10.3390/ijms19113425 30388784 PMC6275017

[B56] Tsuboi M. Kondo K. Soejima S. Kajiura K. Kawakita N. Toba H. (2020). Chromate exposure induces DNA hypermethylation of the mismatch repair gene MLH1 in lung cancer. Mol. Carcinog. 59 (1), 24–31. 10.1002/mc.23125 31579968

[B57] Vitale-Brovarone C. Verné E. Robiglio L. Martinasso G. Canuto R. A. Muzio G. (2008). Biocompatible glass-ceramic materials for bone substitution. J. Mater Sci. Mater Med. 19 (1), 471–478. 10.1007/s10856-006-0111-0 17607523

[B58] Voulgaridou G-P. Tsochantaridis I. Mantso T. Franco R. Panayiotidis M. I. Pappa A. (2017). Human aldehyde dehydrogenase 3A1 (ALDH3A1) exhibits chaperone-like function. Int. J. Biochem. Cell Biol. 89, 16–24. 10.1016/j.biocel.2017.05.017 28526614

[B59] Wang Z. S. Yang C. F. (2019). Metal carcinogen exposure induces cancer stem cell-like property through epigenetic reprograming: A novel mechanism of metal carcinogenesis. Seminars Cancer Biol. 57, 95–104. 10.1016/j.semcancer.2019.01.002 PMC6625953 30641125

[B60] Welling R. Beaumont J. J. Petersen S. J. Alexeeff G. V. Steinmaus C. (2015). Chromium VI and stomach cancer: A meta-analysis of the current epidemiological evidence. Occup. Environ. Med. 72 (2), 151–159. 10.1136/oemed-2014-102178 25231674

[B61] Wilbur S. Abadin H. Fay M. Yu D. Tencza B. Ingerman L. (2012). “Agency for toxic substances and disease registry (ATSDR) toxicological profiles,” in Toxicological profile for chromium (Atlanta (GA): Agency for Toxic Substances and Disease Registry). 24049864

[B62] Wu F. Sun H. Kluz T. Clancy H. A. Kiok K. Costa M. (2012). Epigallocatechin-3-gallate (EGCG) protects against chromate-induced toxicity *in vitro* . Toxicol. Appl. Pharmacol. 258 (2), 166–175. 10.1016/j.taap.2011.10.018 22079256 PMC3259276

[B63] Wu S. Hivert M. F. Cardenas A. Zhong J. Rifas-Shiman S. L. Agha G. (2017). Exposure to low levels of lead *in utero* and umbilical cord blood DNA methylation in Project viva: An epigenome-wide association study. Environ. Health Perspect. 125 (8), 087019. 10.1289/EHP1246 28858830 PMC5783669

[B64] Xie H. Wise S. S. Holmes A. L. Xu B. Wakeman T. P. Pelsue S. C. (2005). Carcinogenic lead chromate induces DNA double-strand breaks in human lung cells. Mutat. Res. 586 (2), 160–172. 10.1016/j.mrgentox.2005.06.002 16112599 PMC4136752

[B65] Xu J. Zhao M. Pei L. Zhang R. Liu X. Wei L. (2018). Oxidative stress and DNA damage in a long-term hexavalent chromium-exposed population in north China: A cross-sectional study. BMJ Open 8 (6), e021470. 10.1136/bmjopen-2017-021470 PMC6020989 29950470

[B66] Xu Y. Wang L. Zhu J. Jiang P. Zhang Z. Li L. (2021). Chromium induced neurotoxicity by altering metabolism in zebrafish larvae. Ecotoxicol. Environ. Saf. 228, 112983. 10.1016/j.ecoenv.2021.112983 34781135

[B67] Yong-Gang T. Peng-Cheng M. Mian-Biao C. Chu-Shan H. Li-Juan Z. Yun-Jiang Y. (2019). Pollution characteristics and potential health risk assessment of heavy metals in household dusts around ferroalloy factory. J. Agric. Resour. Environ. 36 (6), 829. 10.13254/j.jare.2018.0253

[B68] Yuan W. Yang N. Li X. (2016). Advances in understanding how heavy metal pollution triggers gastric cancer. BioMed Res. Int. 2016, 7825432. 10.1155/2016/7825432 27803929 PMC5075591

[B69] Zeng Z. Huo X. Zhang Y. Hylkema M. N. Wu Y. Xu X. (2019). Differential DNA methylation in newborns with maternal exposure to heavy metals from an e-waste recycling area. Environ. Res. 171, 536–545. 10.1016/j.envres.2019.01.007 30763874

[B70] Zhao M. Ge X. Xu J. Li A. Mei Y. Yin G. (2022). Association between urine metals and liver function biomarkers in northeast China: A cross-sectional study. Ecotoxicol. Environ. Saf. 231, 113163. 10.1016/j.ecoenv.2022.113163 35030523

[B71] Zhao M. Xu J. Li A. Mei Y. Ge X. Liu X. (2020). Multiple exposure pathways and urinary chromium in residents exposed to chromium. Environ. Int. 141, 105753. 10.1016/j.envint.2020.105753 32417613

